# Withdrawal of the United States from the World Health Organization (WHO): A Microaggression Against the Developing World and a Self-Inflicted Global Setback

**DOI:** 10.7759/cureus.89672

**Published:** 2025-08-09

**Authors:** Muhammad Hamza Shah

**Affiliations:** 1 Department of Internal Medicine, Antrim Area Hospital, Antrim, GBR; 2 School of Medicine, Queen's University Belfast, Belfast, GBR; 3 Centre for Anatomy, The University of Edinburgh, Edinburgh, GBR

**Keywords:** global health security, healthcare equity, health diplomacy, public health infrastructure, public health policy

## Abstract

The Trump administration's withdrawal from the World Health Organization (WHO) marked a breakdown in global health cooperation. Framed as a nationalist assertion of sovereignty, the move undermined international solidarity during a global pandemic and reflected a broader retreat from multilateral responsibility. This editorial contends that the decision was not merely administrative but ideological, reducing health to a tool of political posturing and isolationist rhetoric. The impact on low- and middle-income countries reliant on WHO support highlights the real-world consequences of such disengagement. What is at stake is the future of global health security and the integrity of international public health systems.

## Editorial

The recent decision by the United States to withdraw from the World Health Organization (WHO) marks a significant departure from decades of American leadership in global health. At the heart of this geopolitical maneuver lies a tension between national sovereignty and global health obligations. The United States has long been the principal financier of the WHO, contributing approximately 15% of its budget in recent years, a financial commitment that granted it significant sway in shaping global health policy [1]. Trump's rationale for withdrawing funds echoes his broader "America First" ideology, which prioritizes national interest over multilateral engagement. 

At its core, the withdrawal of the United States from the WHO is an act of disregard for the realities of the developing world. The WHO is more than just an international body; it is a lifeline for countries with inadequate healthcare infrastructures. By stripping away US funding, the Trump administration has shown an alarming lack of sensitivity to the global disparities in healthcare access and delivery [2]. While the United States continues to operate as the most significant national contributor to international security and health programs, the growing inequities across the world demand shared responsibility and cooperative frameworks. The impact of this decision is particularly acute in low- and middle-income countries, which collectively house 75% of the world's population and 62% of the world's poor [3]. For these nations, the WHO's programs often represent their sole access to essential health services. The absence of support from the United States threatens to dismantle critical health initiatives, leaving these populations vulnerable to preventable diseases and health crises. This decision is not just a withdrawal of financial support; it is a message of abandonment to the very countries that rely on the WHO to bridge the gaps in their own public health systems [4]. It is a direct affront to the dignity and survival of these populations, highlighting an indifference to their needs. Consequently, the shift in priorities away from global health collaboration towards self-preservation and populist agendas is not merely a political miscalculation; it is a microaggression, a dismissal of the developing world's right to participate in the global health dialogue on equal terms.

The decision by the United States to withdraw from the WHO can be viewed not only as a political maneuver but also as a deeply consequential self-inflicted wound that undermines both global stability and its own national interests. At the core of this shift is an ideological commitment to reducing international obligations and prioritizing national interests, often framed as a form of "America First" nationalism [5]. However, this approach fails to account for the interconnected nature of global health and the implications of disregarding collaborative efforts in favor of isolationism. Diseases, both emerging and re-emerging, know no boundaries. The notion that a nation can shield itself from global health threats is an illusion, one that history, particularly the COVID-19 pandemic, has debunked in the wake of successful collaborative efforts [6]. A health crisis in one part of the world can swiftly ripple across borders, as we have seen with the rapid spread of infectious diseases in a globally interconnected environment.

By disengaging from the WHO, the United States may isolate itself from the very global networks and frameworks that enable collective action in response to health crises. The absence of coordinated, multinational efforts often means that health challenges are not just exacerbated in low- and middle-income countries but inevitably come back to impact wealthier nations as well. The COVID-19 pandemic illustrated the importance of a unified approach, with countries that maintained strong international collaborations facing fewer challenges in managing and containing outbreaks [7]. The fragmented, often reactive approach seen in the United States highlighted the limits of national self-sufficiency in the face of global health emergencies, further solidifying the need for multilateral institutions like the WHO that can facilitate swift, unified responses.

Furthermore, by withdrawing from the WHO, the United States could lose a powerful platform for shaping the international health agenda and exerting influence on issues critical to its interests, such as vaccine distribution, intellectual property, and health equity [8]. The WHO's capacity to convene and bring together nations to address urgent global health challenges is unmatched. Without American leadership at the table, other global players, particularly China, are poised to fill the vacuum. China's increasing involvement in global health initiatives, despite its own public health challenges, positions it as a rising leader in areas such as health infrastructure, global vaccine diplomacy, and disease surveillance [9,10]. This shift not only undermines American influence but also sets the stage for a recalibration of international health norms that may not align with US values or interests.

At the same time, Trump's withdrawal is not just a geopolitical shift but also a reflection of deeper ideological currents within the US political landscape. In some ways, the politicization of global health is a tragic irony, for health, by its very nature, transcends political ideologies. The pursuit of health equity, offering every person, regardless of background or political affiliation, the opportunity for a healthy life, is one of the most unifying goals of human civilization. Yet, the moment we allow our differences in ideology to shape our approach to this fundamental issue, we risk undermining the universal values that should guide our health decisions. What, then, is the future of global health when the fight for access to care becomes caught up in a battle over which values and worldviews should prevail? The withdrawal of the United States from the WHO, in this context, represents not just a rejection of an international body but a broader retreat from a vision of global solidarity and shared responsibility that is necessary to confront the vast and interconnected challenges of health in the 21st century. The implications of this ideological standoff reach far beyond politics. They speak to the very nature of how we view health as a fundamental right, a right that should be afforded to all individuals, regardless of where they live or the political landscape that surrounds them. The question, ultimately, is whether we can continue to nurture a global health system that operates on the principles of equity, justice, and solidarity or whether we will allow these ideals to be eroded by the very ideologies that are supposed to serve us all.
